# Clinical Determinants of 30-Day Mortality in Candidemia: Antifungal Susceptibility and Treatment Patterns in a 10-Year Cohort

**DOI:** 10.3390/antibiotics15050438

**Published:** 2026-04-28

**Authors:** İnci Yılmaz Nakir, Esra Zerdali, Selen Aksu, Mustafa Yıldırım

**Affiliations:** Department of Infectious Diseases and Clinical Microbiology, Haseki Training and Research Hospital, İstanbul 34270, Türkiye; esrayerlikaya@gmail.com (E.Z.); selen.aksu.52@gmail.com (S.A.);

**Keywords:** antifungal susceptibility, antifungal therapy, *Candida* species, candidemia, clinical predictors, 30-day mortality

## Abstract

**Objective:** This study aimed to identify clinically modifiable and readily accessible predictors of 30-day mortality in a 10-year candidemia cohort and to assess temporal changes in *Candida* species distribution. **Methods:** We retrospectively evaluated 391 hospitalized adults with positive blood cultures for *Candida* spp. between January 2015 and March 2025. Only the first candidemia episode was included. Demographic characteristics, comorbidities, risk factors, laboratory parameters, antifungal therapy, and outcomes were recorded. Species identification was performed using conventional methods and the VITEK 2 system. Factors associated with 30-day mortality were analyzed using univariate and multivariate logistic regression models. **Results:** The mean age was 64.5 ± 17.7 years, and 56.3% of patients were male. Most patients (68.8%) were managed in the intensive care unit, and the 30-day mortality rate was 54%. Non-*albicans Candida* species accounted for 62.7% of isolates, with an increasing trend over time, particularly for *Candida glabrata*. Fluconazole susceptibility was 79%. In univariate analysis, advanced age, solid tumors, invasive mechanical ventilation, leukocytosis, thrombocytopenia, septic shock, intensive care unit admission, and failure to remove the central venous catheter were associated with mortality. Multivariate analysis identified advanced age, intensive care unit admission, septic shock, failure to remove the central venous catheter, leukocytosis, and thrombocytopenia as independent predictors of 30-day mortality. **Conclusions:** Candidemia remains a life-threatening infection with high mortality. Central venous catheter management and simple hematological parameters, particularly white blood cell and platelet counts, provide practical tools for early risk stratification. Although the rising prevalence of non-*albicans Candida* species may require updates in empirical therapy, prompt source control remains essential to improve survival.

## 1. Introduction

Candidemia is an important clinical condition that causes serious morbidity and mortality among hospital-acquired bloodstream infections. Recent data show that the annual prevalence can reach 7 per 100,000 [1,2]. Mortality rates vary across a wide range. Major risk factors include advanced age, shock or sepsis, and high organ failure scores. Intensive care admission, immunosuppression, and the presence of central venous catheters are also significant factors. Additionally, total parenteral nutrition and widespread antibiotic use are among the primary risk factors [3]. Delayed antifungal therapy has a significant negative impact on survival. Early and appropriate treatment plays a critical role in reducing mortality [4].

The virulence capacity and antifungal resistance profiles of *Candida* species strongly influence clinical outcomes. Infections caused by multidrug-resistant species such as *Candida auris* are associated with higher mortality rates [5]. Several studies have evaluated biomarkers such as β-D-glucan, mannan/anti-mannan, procalcitonin, and other inflammatory markers for diagnosis and prognosis. However, these biomarkers alone do not provide sufficient accuracy. They are more meaningful when interpreted together with clinical parameters [6,7,8,9,10,11].

Accurate identification of clinical and laboratory predictors of mortality in candidemia is essential for early risk stratification and guiding treatment strategies. This study aimed to evaluate clinical, laboratory, and microbiological predictors of 30-day mortality in a large candidemia cohort between January 2015 and March 2025. The detailed assessment of temporal changes in *Candida* species distribution over this decade makes this work one of the few comprehensive studies that address both long-term epidemiological trends and prognostic indicators.

## 2. Results

### Baseline Characteristics

**Figure 1:**
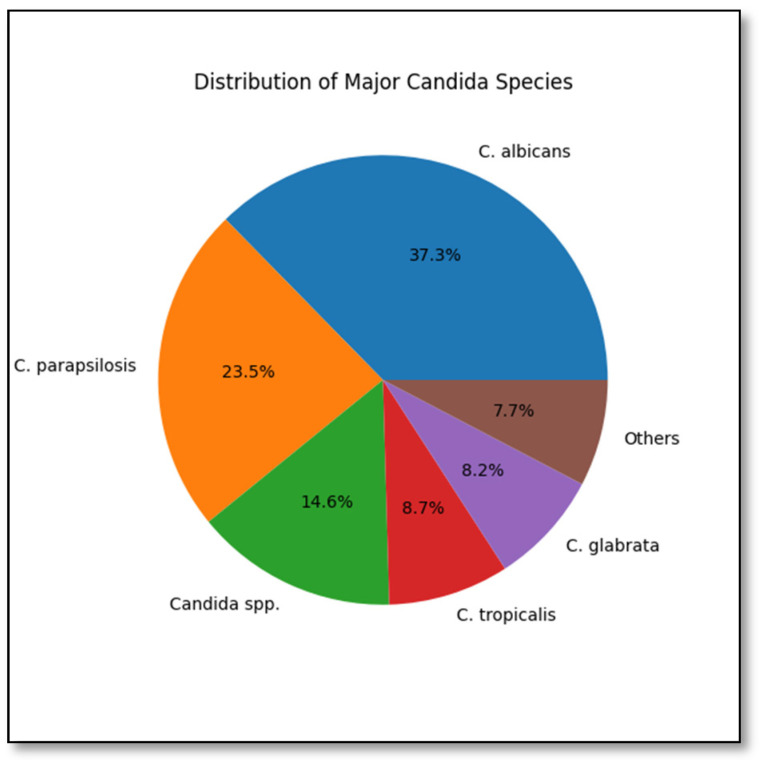
Distribution of major *Candida* species in the study cohort. The most frequently identified species were *Candida albicans*, *C. parapsilosis*, *Candida* spp., *C. tropicalis*, and *C. glabrata*. Less common species were grouped under “Others”.

Among the cases included in the study, 43.7% were female, and 56.3% were male. The mean age was 64.5 ± 17.7 years. Most patients (93.1%) were Turkish citizens. Diabetes mellitus was the most common comorbidity (65.2%). Broad-spectrum antibiotic use was present in 95% of the cases. The mean hospital stay for patients diagnosed with candidemia was 28.4 ± 23.7 days. Additionally, 68.8% of the patients were followed in the intensive care unit. A second candidemia attack occurred in 1.5% of the cases. Concomitant bacteremia was detected in 68%. Ocular involvement was found in 23 patients who underwent eye examinations. Cardiac involvement was identified in 1% of those who underwent echocardiography. Regarding species distribution, non-*albicans Candida* species were dominant at a rate of 62.7%. The distribution of *Candida* species is summarized in Figure 1. Fluconazole susceptibility was 79%. Initial antifungal therapy was frequently started with azole group drugs (72.6%). The compatibility rate of empirical treatment with culture results was 86%. The 30-day mortality rate was 54% (Table 1).

Patients were therefore categorized according to 30-day mortality, defined as all-cause mortality within 30 days of the index positive blood culture: those who died within 30 days (30-day mortality group) and those who survived (survivor group). In the 30-day mortality group, the mean age was significantly higher than in the survivor group (*p* < 0.05). Hypertension, the presence of solid cancer, and the use of invasive mechanical ventilation were also significantly more frequent in this group (*p* < 0.05). No significant differences were found between the groups regarding gender or nationality (*p* > 0.05). Similarly, diabetes mellitus and chronic heart or kidney diseases did not show significant differences. Hematological malignancy, use of immunosuppressive drugs or steroids, and pancreatitis were not significantly different. There were also no significant differences in chronic liver disease, total parenteral nutrition, or history of abdominal surgery. The presence of urinary or central catheters, history of hemodialysis, and use of broad-spectrum antibiotics also did not differ significantly between the groups (*p* > 0.05) (Table 2).

In the mortality group, white blood cell count, neutrophil count, C-reactive protein, urea, creatinine, AST, and procalcitonin levels were significantly higher (*p* < 0.05). Platelet levels were significantly lower in the mortality group (*p* < 0.05). No significant differences were observed between groups in hemoglobin, lymphocyte count, or ALT levels (*p* > 0.05) (Table 2).

When other parameters were evaluated, the hospital stay duration at the time of candidemia was shorter in the mortal group (*p* < 0.05). The rate of second episodes and the rate of catheter replacement were also lower in the mortal group compared to the non-mortal group (*p* < 0.05). Intensive care unit admission, septic shock, and the presence of COVID-19 were significantly higher in the mortal group (*p* < 0.05). Additionally, 14-day mortality was significantly higher in this group (*p* < 0.05). No significant differences were found between the two groups regarding episode type or concomitant bacteremia (*p* > 0.05). Similarly, the distribution of *Candida* species and the presence of endocarditis or ocular involvement did not differ. Antifungal treatment options, fluconazole susceptibility, and the rate of death before receiving treatment were similar. Finally, there was no significant difference in the appropriateness of empirical therapy between the groups (*p* > 0.05) (Table 3).

In the univariate logistic regression analysis, age, hypertension, and solid tumor/chemotherapy were associated with 30-day mortality (*p* < 0.05). Invasive mechanical ventilator use, white blood cell count, and neutrophil count were also significantly related to mortality. Additionally, platelet levels, C-reactive protein, urea, and AST were found to be associated with clinical outcomes. Other significant factors included the duration of hospital stay at the time of candidemia, ward type, and the presence of septic shock. Lack of catheter removal and the presence of COVID-19 were also associated with 30-day mortality (*p* < 0.05). No significant relationship was observed regarding procalcitonin and creatinine levels (*p* > 0.05).

In the multivariate analysis, several factors were identified as independent predictors of 30-day mortality (*p* < 0.05). These included age, white blood cell count, and platelet levels. The duration of hospital stay at the time of candidemia and admission to the intensive care unit were also independent markers. Additionally, the presence of septic shock and the failure to replace the catheter significantly predicted mortality (*p* < 0.05). Detailed results are presented in Table 4.

In the comparison based on *Candida* species, several differences were observed. Procalcitonin levels and the rate of concomitant bacteremia were significantly lower in the non-*albicans Candida* group compared to the *albicans* group (*p* < 0.05). The rate of catheter removal was also lower in the non-albicans Candida group compared to the albicans group (*p* < 0.05). Conversely, echinocandin treatment and the presence of COVID-19 were significantly higher in the non-*albicans Candida* group (*p* < 0.05). No significant differences were found regarding other parameters (*p* > 0.05) (Table 5 and Table 6).

When the temporal distribution was examined, the rates of *C. albicans* and *C. famata* decreased between 2020 and 2025 (*p* < 0.05). In contrast, the rates of *Candida* spp. and *C. glabrata* increased during the same period (*p* < 0.05). The distribution of other species did not show a significant difference between the periods (*p* > 0.05) (Table 7).

## 3. Discussion

In this study, the 30-day mortality rate was 54.5%. This high rate is consistent with the 30–60% range reported in the literature [12,13]. This finding indicates a high-risk profile in our cohort. In the multivariate analysis, advanced age, intensive care unit admission, and septic shock were identified as independent prognostic markers. These results align with data from large-scale studies and meta-analyses [14,15,16]. Such reports emphasize that candidemia significantly increases mortality, especially in severely ill patients.

The evaluation of laboratory parameters revealed significant leukocytosis and neutrophilia in the mortal group. These patients also had increased CRP, procalcitonin (PCT), and urea/creatinine levels. These findings are consistent with studies reporting that systemic inflammation and renal dysfunction are associated with poor clinical outcomes in candidemia [17,18]. Notably, a low platelet level was identified as an independent prognostic marker. Thrombocytopenia has recently been defined as one of the biomarkers most strongly associated with a poor prognosis in candidemia. Our study clearly supports this relationship [17,18]. These parameters are easily accessible and effective tools. They can be used to identify high-risk patients early and to plan aggressive management strategies in intensive care.

In this study, the failure to remove the central venous catheter (CVC) was independently associated with mortality. The positive effect of catheter removal on prognosis was also identified. This finding is consistent with current evidence and international guidelines reporting that early catheter removal reduces mortality in catheter-related candidemia [19,20]. The decisive role of CVC management is clearly seen in the multivariate analysis of our study. This is particularly relevant for patients in intensive care units who require invasive procedures and total parenteral nutrition. The independent effect of timely catheter removal on clinical outcomes highlights the critical importance of catheter strategies in candidemia management once again.

Taken together, these findings highlight that advanced age, ICU admission, septic shock, failure to remove the central venous catheter, leukocytosis, and thrombocytopenia constitute the core determinants of 30-day mortality in candidemia. These findings provide a practical framework for early risk stratification and targeted clinical management.

The distribution of species showed that the rate of non-*albicans Candida* reached 62.7%. The increase in non-*albicans Candida* species between 2020 and 2025 is consistent with the epidemiological shift reported globally [21,22]. Increased invasive procedures and prolonged ICU stays during the COVID-19 pandemic played a key role in this trend. The rise in broad-spectrum antibiotic and steroid use also contributed to this increase. In our study, a significant increase was observed in unidentified *Candida* isolates during the 2020–2025 period. Routine identification of *C. auris* began at our center in 2025. Limitations in confirmatory methods in previous years suggest that this species might have been overlooked in some cases. Therefore, the increase in unidentified isolates is noteworthy. It remains uncertain whether this indicates early *C. auris* circulation. This trend emphasizes the importance of closely monitoring local epidemiological data and susceptibility patterns. Fluconazole susceptibility was 79% in our cohort. While this is a positive finding, the increase in non-*albicans Candida* species necessitates the updating of empirical treatment protocols.

Regarding treatment approaches, azole group antifungals were more frequently preferred as the initial choice. However, the use of echinocandins was higher in severe cases and when non-*albicans Candida* species were suspected. This approach is consistent with current guidelines, which recommend echinocandins as the first-line option for candidemia cases, particularly in critically ill patients [23,24].

During a significant part of the study period, the Health Implementation Communiqué (SUT) in our country included restrictions on the empirical use of echinocandins. This situation may have influenced our high rate of empirical fluconazole use. The restriction in question was removed around 2020. Since then, clinical access to echinocandins has become easier. Therefore, the frequent preference for fluconazole in empirical treatment reflects national reimbursement policies rather than our center’s clinical choice. In accordance with guideline recommendations, the use of echinocandins in severe patients is expected to increase following this regulation.

The high accuracy rate of 86% for empirical treatment according to culture results indicates that candidemia management at our center is generally successful. This finding serves as an important quality indicator. The association between COVID-19 positivity and mortality is consistent with the literature regarding the pandemic period. In COVID-19 patients, the frequency of invasive procedures and the need for ICU admission increased significantly. Furthermore, the use of broad-spectrum antibiotics and steroids rose markedly. These are the primary factors that increase both the risk of candidemia and mortality [25,26].

In our study, hypertension, the requirement for invasive mechanical ventilation, and the presence of a solid tumor/chemotherapy were associated with a mortal course. These findings parallel previous studies. The finding that chronic renal failure was not associated with mortality supports the results of a study conducted in our country. Although different results are reported in the literature on this subject, there is no definitive consensus [27,28,29].

In addition, part of our study period overlapped with the COVID-19 pandemic. During the pandemic, increased ICU admissions, invasive procedures, central venous catheter use, and corticosteroid therapy may have contributed to the development of candidemia [28,30]. A large systematic review and meta-analysis including COVID-19 patients reported a significant prevalence of candidemia among critically ill patients and highlighted the high associated mortality. These findings suggest that the COVID-19 pandemic may have had a potential impact on the epidemiology and clinical outcomes of candidemia [31].

Overall, this study has comprehensively demonstrated the clinical, laboratory, and management-based predictors of 30-day mortality due to candidemia in a large, 10-year cohort. Several parameters had independent prognostic significance. These included advanced age, thrombocytopenia, and admission to the intensive care unit. Septic shock and central venous catheter management were also critical markers. These findings provide a vital framework for early risk assessment and the correct direction of treatment strategies. The identification of easily accessible laboratory markers, such as WBC and platelets, as independent predictors is a key finding. This can contribute to the development of rapid and applicable risk classification in clinical practice. Furthermore, the independent effect of timely central venous catheter removal on mortality clearly demonstrates the importance of quality improvement initiatives regarding catheter management, especially in intensive care populations.

The significant increase in non-albicans species and the rise in unidentified isolates in recent years indicate new epidemiological dynamics in the post-COVID-19 era. These trends highlight the necessity of regularly updating local surveillance and antifungal susceptibility patterns. This study is among the few comprehensive cohorts in the literature that evaluate long-term epidemiological shifts alongside clinical and laboratory markers. The changes in non-albicans prevalence and the increase in unidentified isolates provide critical data. These findings serve as a warning for updating local treatment algorithms and strengthening infection control policies.

Future studies should focus on identifying high-risk patients at an earlier stage and improving preventive strategies for candidemia. The development of risk prediction models based on clinical and laboratory parameters may support early diagnosis and timely intervention. In addition, machine learning-based approaches may further enhance risk stratification and clinical decision-making.

This study has several limitations. First, its retrospective design and single-center setting may limit the generalizability of the findings and introduce potential selection and information bias. Additionally, full standardization of microbiological culture and antifungal susceptibility testing methods could not be ensured throughout the study period. The lack of routine molecular identification may have affected the accuracy of species-level classification in some isolates. Furthermore, some clinical variables and diagnostic evaluations, such as ophthalmological examinations, were not available for all patients, which may have influenced the assessment of certain complications.

Despite these limitations, this study has several strengths. It evaluates long-term epidemiological trends over a 10-year period and identifies clinically relevant prognostic markers. Moreover, the findings have practical implications for centers with similar patient populations.

## 4. Materials and Methods

### 4.1. Ethical Approval

The study was approved by the Clinical Research Ethics Committee of SBU Haseki Training and Research Hospital. The approval date is 25 June 2025, and the decision number is 109-2025. The study was conducted in accordance with the Declaration of Helsinki.

### 4.2. Study Design and Population

This study was conducted as a retrospective cohort study at the Department of Infectious Diseases and Clinical Microbiology, SBÜ Haseki Training and Research Hospital. Between January 2015 and March 2025, adult patients (≥18 years) who were hospitalized and had at least one blood culture positive for *Candida* spp. were included. For each patient, only the first episode of candidemia (index episode) was analyzed. In patients with multiple episodes, subsequent attacks were excluded from the analysis.

### 4.3. Inclusion and Exclusion Criteria

Adult patients aged 18 years or older, hospitalized, and with blood cultures positive for *Candida* species were included in the study. Only cases with complete access to clinical and laboratory data were analyzed. Patients younger than 18 years were excluded. Outpatients and patients referred from external centers were also excluded. Blood cultures with growth of yeasts or molds other than *Candida* species were not included. Cases with polymicrobial growth that were not clinically consistent with candidemia were excluded. Patients with missing data were also excluded from the study.

### 4.4. Definitions

Candidemia was defined as the growth of *Candida* spp. in at least one blood culture in a patient with clinical signs and symptoms. Candidemia detected within 30 days of the first isolation was considered the first episode. Cases occurring more than 30 days after the first isolation with negative blood cultures in between were evaluated as a second episode. Only the first episode of candidemia was included in the study. All-cause deaths occurring within 30 days following the date of *Candida* isolation in blood culture were defined as 30-day mortality. Steroid use was defined as systemic corticosteroid administration within the 7 days preceding the onset of candidemia. Ocular involvement was defined as the presence of chorioretinitis or endophthalmitis detected during ophthalmologic examination. Death before treatment was defined as patients who died after blood cultures were obtained but before empirical antifungal therapy could be initiated, either before the culture results became available or before treatment initiation.

### 4.5. Data Sources and Data Collection

Patient data were retrospectively obtained from the hospital information management system. Recorded variables included demographic characteristics such as age and sex. Sex was recorded as biological sex (male/female) based on the information documented in the patients’ medical records. The ward or intensive care unit of hospitalization was also documented. Underlying diseases were recorded, including diabetes mellitus, malignancy, cardiovascular disease, renal disease, and liver disease. Risk factors were identified for each case. These included mechanical ventilation, central venous catheter (CVC) use, total parenteral nutrition (TPN), chemotherapy, use of broad-spectrum antibiotics, hemodialysis, systemic corticosteroid therapy, abdominal surgery, neutropenia, and concomitant bacteremia. Laboratory parameters were also retrieved. The date of candidemia diagnosis was defined as the index day. Each patient was followed for 30 days after diagnosis. Mortality and clinical outcomes during this period were recorded. In our cohort, fluconazole was the only azole antifungal agent used for treatment.

### 4.6. Microbiological Investigations

Blood samples sent for culture were incubated at 35 °C for up to 7 days using the BacT/Alert 3D automated blood culture system (bioMérieux, Marcy l’Étoile, France). Bottles flagged as positive were subcultured onto 5% Sheep Blood Agar (Oxoid, Thermo Fisher Scientific, Basingstoke, UK), Sabouraud Dextrose Agar (SDA), and CHROMagar Candida (Oxoid Brilliance Candida Agar) and incubated aerobically at 37 °C for 24–48 h.

Yeast growth was confirmed by Gram staining. Preliminary identification was performed using colony morphology, germ tube testing, and conventional phenotypic methods. Species-level identification was carried out using the VITEK 2 Compact system (YST ID card, bioMérieux, Marcy l’Étoile, France).

For antifungal susceptibility testing, Candida isolates were adjusted to a 2.0 McFarland standard (range 1.8–2.2) in 0.45% sterile NaCl according to the manufacturer’s instructions and analyzed using the VITEK 2 Compact system (AST YS08 card, bioMérieux, Marcy l’Étoile, France). Results were interpreted according to the clinical breakpoints and epidemiological cutoff values (ECOFFs) defined by the Clinical and Laboratory Standards Institute (CLSI).

In this study, antifungal susceptibility refers to in vitro susceptibility testing of Candida isolates rather than clinical response to antifungal therapy. Isolates not identified to the species level were reported as *Candida* spp. and analyzed accordingly. All blood culture and antifungal susceptibility tests were performed in the central microbiology laboratory of our hospital, and the results were retrospectively retrieved from the hospital information system.

### 4.7. Laboratory Analyses

Complete blood counts, biochemical parameters, and procalcitonin levels were analyzed in the central laboratory of the institution. All analyses were performed using original reagents supplied by the manufacturers.

### 4.8. Statistical Analysis

Descriptive statistics are reported as mean ± standard deviation and/or median (minimum–maximum) for continuous variables and as numbers (percentages) for categorical variables. The distribution of variables was assessed using the Kolmogorov–Smirnov and Shapiro–Wilk tests. For comparisons between two groups, the Mann–Whitney U test was used for non-normally distributed continuous variables, while the chi-square test or Fisher’s exact test was applied for categorical variables, as appropriate. A two-sided *p* value < 0.05 was considered statistically significant. All analyses were performed using SPSS software, version 27.0 (IBM Corp., Armonk, NY, USA).

Variables associated with the primary outcome of 30-day mortality were first evaluated using univariable logistic regression analysis. Variables with a *p* value < 0.10 and/or those considered clinically relevant were subsequently included in a multivariable logistic regression model. A forward stepwise likelihood ratio (Forward LR) approach was applied in the multivariable analysis, and results are presented as odds ratios (ORs) with 95% confidence intervals (CIs) and *p* values.

## 5. Conclusions

This study clearly demonstrates the key clinical and laboratory markers associated with 30-day mortality in patients with candidemia. Advanced age, leukocytosis, and thrombocytopenia were identified as independent and strong predictors of mortality. Septic shock, intensive care unit admission, and failure to remove the central venous catheter were also significant independent risk factors. In addition, the increasing proportion of non-*albicans Candida* species highlights the need for continuous monitoring of local epidemiology and antifungal susceptibility patterns.

Our findings underscore the critical importance of early risk stratification, timely source control through catheter removal, and antifungal therapy guided by current resistance profiles in the management of candidemia. Collectively, these results provide a practical framework to support the development of evidence-based and standardized clinical protocols, particularly in intensive care settings.

The routinely available and structured predictors identified in this study provide a suitable foundation for future machine learning-based risk prediction models in candidemia, with the potential to support early clinical decision-making.

## Figures and Tables

**Table 1:** Baseline demographic, clinical, and laboratory characteristics of patients with candidemia.

Parameters		Minimum–Maximum			Median	(Mean ± SD)/ n-%		
Age		18.0	-	100.0	68.0	64.5	±	17.7
Sex	Female					171		43.7%
	Male					220		56.3%
Nationality	Republic of Türkiye					364		93.1%
	Other					27		6.9%
**Comorbidities**								
Diabetes Mellitus						255		65.2%
Hypertension						228		58.3%
Chronic Heart Disease						158		40.4%
Chronic Kidney Disease						128		32.7%
Solid Malignancy/Chemotherapy Status						122		31.2%
Use of Immunosuppressive Drugs						113		28.9%
Chronic Renal Failure						88		22.5%
Steroid Use						43		11.0%
Hematologic Malignancy/Chemotherapy Status						22		5.6%
Chronic Liver Disease/Cirrhosis/Ascites						22		5.6%
Acute Pancreatitis						14		3.6%
HIV Positivity						6		1.5%
**Risk Factors**								
Use of Broad-Spectrum Antibiotics						372		95.1%
Urinary Catheter						356		91.0%
Central Venous Catheter						331		84.7%
History of Invasive Mechanical Ventilation						298		76.2%
History of Abdominal Surgery						157		40.2%
History of Hemodialysis						147		37.6%
Total Parenteral Nutrition						133		34.0%
**Laboratory Parameters**								
White Blood Cell Count (10^3^/µL)		0.1	-	65.0	10.6	12.3	±	7.9
Neutrophil Count (10^3^/µL)		0.4	-	80.0	8.6	10.5	±	8.3
Lymphocyte Count (10^3^/µL)		0.1	-	15.0	1.0	1.3	±	1.5
Hemoglobin		5.0	-	16.3	9.0	9.1	±	1.7
Platelet Count (10^3^/µL)		5.0	-	800.0	184.0	201.2	±	135.1
C-Reactive Protein (mg/L)		8.0	-	479.0	144.0	161.4	±	96.8
Procalcitonin (µg/L)		0.0	-	262.0	2.4	8.6	±	25.8
Urea		1.7	-	372.0	61.0	81.4	±	62.5
Creatinine (mg/dL)		0.0	-	11.1	1.0	1.6	±	1.7
Alanine Aminotransferase (ALT)		3.0	-	8909.0	24.0	81.4	±	482.2
Aspartate Aminotransferase (AST)		1.0	-	8709.0	34.0	114.3	±	519.3
**Other Parameters**								
Length of Hospital Stay at Onset of Candidemia		1.0	-	180.0	23.0	28.4	±	23.7
Hospital Ward		Internal Medicine				79		20.2%
		Surgical				43		11.0%
		Intensive Care Unit				269		68.8%
Second Episode		Yes				6		1.5%
		No				385		98.5%
Presence of Concomitant Bacteremia						266		68.0%
Septic Shock						218		55.8%
Candida Albicans/Non-Albicans		Candida Albicans				146		37.3%
		Non-Albicans				245		62.7%
Endocarditis						4		1.0%
Ocular Involvement						23		5.9%
Echinocandin Therapy						16		4.1%
Central Venous Catheter Removal						299		76.5%
Fluconazole		**Not Tested**				28		7.2%
		**Susceptible**				309		79.0%
		**Resistant**				47		12.0%
		**Intermediate**				7		1.8%
Initial Antifungal Therapy		Azole				284		72.6%
		Echinocandin				73		18.7%
		Amphotericin B				3		0.8%
		Death Before Initiation of Treatment				31		7.9%
Appropriateness of Initial Therapy According to Culture Results		Yes				311		86.4%
		No				49		13.6%
Presence of COVID-19		Yes				49		12.5%
		No				342		87.5%
14-Day Mortality						32		8.2%
30-Day Mortality						213		54.5%

Data are presented as mean ± standard deviation (SD) and median (minimum–maximum) for continuous variables and as number (percentage) for categorical variables. Only the first episode of candidemia for each patient was included in the analysis; ALT, alanine aminotransferase; AST, aspartate aminotransferase. Reference ranges: WBC 4–10 × 10^3^/µL; neutrophils 1.78–5.38 × 10^3^/µL; lymphocytes 1–3 × 10^3^/µL; hemoglobin 12–16 g/dL (female) and 13–17 g/dL (male); platelets 150–450 × 10^3^/µL; CRP < 5 mg/L; procalcitonin <0.05 µg/L; urea 17–43 mg/dL; creatinine 0.6–1.2 mg/dL; ALT < 45 U/L; AST < 35 U/L. Reference ranges may vary slightly between laboratories.

**Table 2:** Comparison of demographic, clinical, and laboratory characteristics according to 30-day mortality in patients with candidemia.

Parameters		30-Day Mortality (−) (n:178)					30-Day Mortality (+) (n:213)				*p*-Values	
		(Mean ± SD)/n-%			Median		(Mean ± SD)/n-%			Median		
Age (Mean ± SD)		60.7	±	18.6	63.5		67.7	±	16.2	69.0	**<0.001**	^m^
Sex	Female	75		42.1%			96		45.1%		0.560	χ^2^
	Male	103		57.9%			117		54.9%			
Nationality	Republic of Türkiye	166		93.3%			198		93.0%		0.907	χ^2^
	Other	12		6.7%			15		7.0%			
**COMORBIDITIES**												
Diabetes Mellitus		111		62.4%			144		67.6%		0.278	χ^2^
Hypertension		94		52.8%			134		62.9%		**0.044**	χ^2^
Chronic Heart Disease		64		36.0%			94		44.1%		0.101	χ^2^
Chronic Kidney Disease		53		29.8%			75		35.2%		0.254	χ^2^
Chronic Renal Failure		35		19.7%			53		24.9%		0.218	χ^2^
Solid Malignancy/Chemotherapy		44		24.7%			78		36.6%		**0.011**	χ^2^
Immunosuppressive Therapy		50		28.1%			63		29.6%		0.747	χ^2^
Steroid Use		21		11.8%			22		10.3%		0.644	χ^2^
**RISK FACTORS**												
Total Parenteral Nutrition		59		33.1%			74		34.7%		0.740	χ^2^
History of Abdominal Surgery		73		41.0%			84		39.4%		0.752	χ^2^
Urinary Catheter		160		89.9%			196		92.0%		0.462	χ^2^
Central Venous Catheter		147		82.6%			184		86.4%		0.299	χ^2^
Invasive Mechanical Ventilation		124		69.7%			174		81.7%		**0.005**	χ^2^
History of Hemodialysis		61		34.3%			86		40.4%		0.214	χ^2^
Antibiotic Use		168		94.4%			204		95.8%		0.524	χ^2^
**LABORATORY PARAMETERS**												
White Blood Cell Count (10^3^/µL)		11.0	±	6.4	9.8		13.4	±	8.8	11.7	**0.003**	^m^
Neutrophils (10^3^/µL)		9.3	±	8.1	7.5		11.5	±	8.3	9.8	**<0.001**	^m^
Lymphocyte Count (10^3^/µL)		1.4	±	1.2	1.0		1.3	±	1.7	1.0	0.055	^m^
Hemoglobin		9.1	±	1.6	9.0		9.0	±	1.8	9.0	0.845	^m^
Platelet Count (10^3^/µL)		232.0	±	142.1	217.5		175.8	±	123.7	160.0	**<0.001**	^m^
C-Reactive Protein (mg/L)		144.5	±	92.6	129.5		175.3	±	98.1	165.0	**0.002**	^m^
Procalcitonin (µg/L)		6.3	±	18.3	2.0		10.6	±	30.6	2.9	**0.004**	^m^
Urea		67.8	±	55.9	50.5		92.7	±	65.4	77.0	**<0.001**	^m^
Creatinine (mg/dL)		1.4	±	1.7	0.8		1.7	±	1.7	1.2	**0.005**	^m^
ALT		34.9	±	56.1	19.0		119.9	±	647.8	25.0	0.063	^m^
AST		52.9	±	106.7	33.5		164.9	±	691.8	37.0	**0.030**	^m^

Data are presented as mean ± standard deviation (SD) and median for continuous variables and as number (percentage) for categorical variables. Comparisons between groups were performed using the Mann–Whitney U test for continuous variables and the chi-square test or Fisher’s exact test for categorical variables, as appropriate; ^m^ indicates the Mann–Whitney U test; χ^2^ indicates the chi-square test. Bold values indicate statistical significance (*p* < 0.05). ALT, alanine aminotransferase; AST, aspartate aminotransferase. Only the most common and clinically relevant comorbidities are presented in the table to improve readability and avoid redundancy. (+) indicates patients who died within the specified time period (14 days or 30 days), while (−) indicates patients who survived. Reference ranges: WBC 4–10 × 10^3^/µL; neutrophils 1.78–5.38 × 10^3^/µL; lymphocytes 1–3 × 10^3^/µL; hemoglobin 12–16 g/dL (female) and 13–17 g/dL (male); platelets 150–450 × 10^3^/µL; CRP < 5 mg/L; procalcitonin < 0.05 µg/L; urea 17–43 mg/dL; creatinine 0.6–1.2 mg/dL; ALT < 45 U/L; AST < 35 U/L. Reference ranges may vary slightly between laboratories.

**Table 3:** Clinical characteristics, microbiological findings, and treatment-related variables according to 30-day mortality in patients with candidemia.

OTHER PARAMETERS			30-Day Mortality (−) (n:178)					30-Day Mortality (+) (n:213)				*p*-Values	
			(Mean ± SD)/n-%			Median		(Mean ± SD)/n-%			Median		
**Length of Hospital Stay at Onset of Candidemia**			32.8	±	28.0	27.0		24.7	±	18.6	21.0	**0.015**	^m^
**Hospital Ward**	Internal Medicine		45		25.3%			34		16.0%		**<0.001**	χ^2^
	Surgical		31		17.4%			12		5.6%			
	Intensive Care Unit		102		57.3%			167		78.4%			
**Second Episode**	Yes		6		3.4%			0		0.0%		**0.007**	χ^2^
	No		172		96.6%			213		100%			
**Presence of Concomitant Bacteremia**			129		72.5%			137		64.3%		0.085	χ^2^
**Septic Shock**			66		37.1%			152		71.4%		**<0.001**	χ^2^
**Candida** **Species**	*C. albicans*		72		40.4%			74		34.7%		0.245	χ^2^
	Non-albicans		106		59.6%			139		65.3%			
**Clinical** **Complication**	Endocarditis		3		1.7%			1		0.5%		0.334	χ^2^
	Ocular Involvement		12		6.7%			11		5.2%		χ^2^	χ^2^
**Echinocandin Therapy**			6		3.4%			10		4.7%		0.510	χ^2^
**Central Venous Catheter Removal**			153		86.0%			146		68.5%		**<0.001**	χ^2^
**Fluconazole**													
Susceptible			136		83.4%			173		86.5%		0.660	χ^2^
Resistant			24		14.7%			23		11.5%			
Intermediate			3		1.8%			4		2.0%			
**Initial Antifungal Therapy**													
Azole			125		70.2%			159		74.6%		0.329	χ^2^
Echinocandin			37		20.8%			36		16.9%		0.326	χ^2^
Amphotericin B			2		1.1%			1		0.5%		0.593	χ^2^
**Death Before Treatment**			14		7.9%			17		8.0%		0.966	χ^2^
**Appropriateness of Initial Therapy According to Culture Results**		Yes	142		86.6%			169		86.2%		0.921	χ^2^
		No	22		13.4%			27		13.8%			
**Presence of COVID-19**		Yes	12		6.7%			37		17.4%		**0.002**	χ^2^
		No	166		93.3%			176		82.6%			
**14-Day Mortality**			0		0.0%			32		15.0%		**<0.001**	χ^2^

Data are presented as mean ± standard deviation (SD) and median for continuous variables and as number (percentage) for categorical variables. Comparisons between groups were performed using the Mann–Whitney U test for continuous variables and the chi-square test or Fisher’s exact test for categorical variables, as appropriate; ^m^ indicates the Mann–Whitney U test; χ^2^ indicates the chi-square test. (+) indicates patients who died within the specified time period (14 days or 30 days), while (−) indicates patients who survived.

**Table 4:** Univariate and multivariate logistic regression analysis of factors associated with 30-day mortality in patients with candidemia.

Parameters	Univariate Model					Multivariate Model				
	OR	%95 GA			*p*	OR	%95 GA			*p*
Age	1.023	1.011	-	1.036	**<0.001**	1.019	1.005	-	1.033	**0.006**
Hypertension	0.660	0.440	-	0.989	**0.044**					
Solid Malignancy/Chemotherapy	0.568	0.366	-	0.883	**0.012**					
Invasive Mechanical Ventilation	0.515	0.321	-	0.825	**0.006**					
White Blood Cell Count (10^3^/µL)	1.045	1.015	-	1.075	**0.003**	1.041	1.006	-	1.078	**0.023**
Neutrophils (10^3^/µL)	1.038	1.009	-	1.068	**0.011**					
Platelet Count (10^3^/µL)	0.997	0.995	-	0.998	**<0.001**	0.997	0.995	-	0.999	**0.005**
C-Reactive Protein (mg/L)	1.003	1.001	-	1.006	**0.002**					
Procalcitonin (µg/L)	1.008	0.998	-	1.018	0.136					
Urea	1.007	1.003	-	1.011	**<0.001**					
Creatinine (mg/dL)	1.130	0.994	-	1.285	0.063					
AST	1.002	1.000	-	1.004	**0.018**					
Length of Hospital Stay at Onset of Candidemia	0.985	0.976	-	0.994	**0.001**	0.986	0.976	-	0.996	**0.005**
Hospital Ward	1.600	1.243	-	2.059	**<0.001**	1.353	1.014	-	1.807	**0.040**
Septic Shock	0.236	0.155	-	0.362	**<0.001**	0.338	0.212	-	0.540	**<0.001**
Catheter Removal	2.808	1.683	-	4.688	**<0.001**	2.190	1.223	-	3.924	**0.008**
COVID-19 Diagnosis	0.344	0.173	-	0.682	**0.002**					

Odds ratios (ORs) with 95% confidence intervals (CIs) were calculated using univariate and multivariate logistic regression analyses. Variables with *p* < 0.10 in univariate analysis were included in the multivariate model. Multivariate analysis was performed using the forward likelihood ratio (Forward LR) method. Odds ratios (ORs) < 1 indicate an increased risk of 30-day mortality, as the dependent variable was coded as survival. OR, odds ratio; CI, confidence interval; Bold values indicate statistically significant results (*p* < 0.05).

**Table 5:** Comparison of demographic, clinical, and laboratory characteristics between *Candida albicans* and non-albicans Candida groups.

Parameters		Albicans Candida n/% (n:146)					Non-Albicans Candida n/% (n:245)				*p*-Value	
					Median					Median		
Age (Mean ± SD)		65.5	±	16.5	68.5		64.0	±	18.3	67.0	0.452	^m^
Sex	Female	72		49.3%			99		40.4%		0.086	χ^2^
	Male	74		50.7%			146		59.6%			
Nationality	Republic of Türkiye	137		93.8%			227		92.7%		0.656	χ^2^
	Other	9		6.2%			18		7.3%			
**COMORBIDITIES**												
**Diabetes Mellitus**		96		65.8%			159		64.9%		0.864	χ^2^
**Hypertension**		91		62.3%			137		55.9%		0.214	χ^2^
**Chronic Heart Disease**		67		45.9%			91		37.1%		0.088	χ^2^
**Chronic Kidney Disease**		45		30.8%			83		33.9%		0.533	χ^2^
**Chronic Renal Failure**		38		26.0%			50		20.4%		0.198	χ^2^
**Solid Malignancy/Chemotherapy**		44		30.1%			78		31.8%		0.726	χ^2^
**Use of Immunosuppressive Drugs**		38		26.0%			75		30.6%		0.333	χ^2^
**Steroid Use**		16		11.0%			27		11.0%		0.985	χ^2^
**RISK FACTORS**												
**Total Parenteral Nutrition**		48		32.9%			85		34.7%		0.714	χ^2^
**History of Abdominal Surgery**		64		43.8%			93		38.0%		0.252	χ^2^
**Urinary Catheter**		134		91.8%			222		90.6%		0.695	χ^2^
**Central Venous Catheter**		121		82.9%			210		85.7%		0.451	χ^2^
**Invasive Mechanical Ventilation**		108		74.0%			190		77.6%		0.421	χ^2^
**History of Hemodialysis**		56		38.4%			91		37.1%		0.811	χ^2^
**Broad-Spectrum Antibiotic Use**		138		94.5%			234		95.5%		0.660	χ^2^
**LABORATORY PARAMETERS**												
**White Blood Cell Count (10^3^/µL)**		12.3	±	6.8	11.1		12.3	±	8.5	10.5	0.546	^m^
**Neutrophils (10^3^/µL)**		10.8	±	8.6	9.0		10.4	±	8.0	8.5	0.404	^m^
**Lymphocyte Count (10^3^/µL)**		1.46	±	1.85	1.02		1.27	±	1.22	1.00	0.340	^m^
**Hemoglobin**		9.02	±	1.66	8.90		9.08	±	1.71	9.00	0.574	^m^
**Platelet Count (10^3^/µL)**		205.4	±	133.0	200.0		198.7	±	136.6	180.0	0.503	^m^
**C-Reactive Protein (mg/L)**		167.6	±	95.1	151.5		157.6	±	97.8	141.0	0.232	^m^
**Procalcitonin (µg/L)**		7.3	±	13.2	3.0		9.4	±	31.0	2.2	**0.014**	^m^
**Urea**		81.0	±	58.9	64.5		81.7	±	64.6	60.0	0.826	^m^
**Creatinine (mg/dL)**		1.72	±	1.87	1.09		1.49	±	1.56	0.85	0.228	^m^
**ALT**		73.1	±	250.5	24.0		86.5	±	578.9	24.0	0.968	^m^
**AST**		102.4	±	249.4	34.0		121.4	±	628.5	35.0	0.767	^m^

Data are presented as mean ± standard deviation (SD) and median for continuous variables and as number (percentage) for categorical variables. Comparisons between groups were performed using the Mann–Whitney U test for continuous variables and the chi-square test or Fisher’s exact test for categorical variables, as appropriate; ^m^ indicates the Mann–Whitney U test; χ^2^ indicates the chi-square test. Bold values indicate statistically significant results (*p* < 0.05). ALT, alanine aminotransferase; AST, aspartate aminotransferase *Candida albicans* is written in italics according to taxonomic conventions. Only the most common and clinically relevant comorbidities are presented in the table to improve readability and avoid redundancy. Reference ranges: WBC 4–10 × 10^3^/µL; neutrophils 1.78–5.38 × 10^3^/µL; lymphocytes 1–3 × 10^3^/µL; hemoglobin 12–16 g/dL (female) and 13–17 g/dL (male); platelets 150–450 × 10^3^/µL; CRP < 5 mg/L; procalcitonin < 0.05 µg/L; urea 17–43 mg/dL; creatinine 0.6–1.2 mg/dL; ALT < 45 U/L; AST < 35 U/L. Reference ranges may vary slightly between laboratories.

**Table 6:** Selected clinical, microbiological, and treatment-related characteristics according to Candida species in patients with candidemia.

OTHER PARAMETERS		Albicans Candida n/% (n:146)			Median		Non-Albicans Candida n/% (n:245)			Median	*p*-Value	
**Length of Hospital Stay at Onset of** **Candidemia**		27.1	±	23.7	22.5		29.2	±	23.7	24.0	0.424	^m^
**Hospital Ward**	Internal Medicine	34		23.3%			45		18.4%		0.151	χ^2^
	Surgical	20		13.7%			23		9.4%			
	Intensive Care Unit	92		63.0%			177		72.2%			
**Presence of Concomitant Bacteremia**		109		74.7%			157		64.1%		**0.030**	χ^2^
**Septic Shock**		85		58.2%			133		54.3%		0.449	χ^2^
**Clinical** **Complication**	Endocarditis	3		2.1%			1		0.4%		0.149	χ^2^
	Ocular involvement	10		6.8%			13		5.3%		0.530	χ^2^
**Echinocandin Therapy**		1		0.7%			15		6.1%		**0.009**	χ^2^
**Central venous catheter (CVC) removal**		120		82.2%			179		73.1%		**0.040**	χ^2^
**Fluconazole**												
Susceptible		119		88.8%			190		83.0%		0.320	χ^2^
Resistant		13		9.7%			34		14.8%			
Intermediate		2		1.5%			5		2.2%			
**Death Before Treatment**		12		8.2%			19		7.8%		0.870	χ^2^
**Appropriate Antifungal Therapy?**	Yes	119		81.5%			192		78.4%		0.303	χ^2^
	No	15		10.3%			34		13.9%			
**14-Day Mortality**	(−)	134		91.8%			225		91.8%		0.984	χ^2^
	(+)	12		8.2%			20		8.2%			
**30-Day Mortality**	(−)	72		49.3%			106		43.3%		0.245	χ^2^
	(+)	74		50.7%			139		56.7%			
**Presence of COVID-19**	Yes	9		6.2%			40		16.3%		**0.003**	χ^2^
	No	137		93.8%			205		83.7%			

Data are presented as mean ± standard deviation (SD) and median for continuous variables and as number (percentage) for categorical variables. Comparisons between groups were performed using the Mann–Whitney U test for continuous variables and the chi-square test or Fisher’s exact test for categorical variables, as appropriate; ^m^ indicates the Mann–Whitney U test; χ^2^ indicates the chi-square test. Bold values indicate statistically significant results (*p* < 0.05). *Candida albicans* is written in italics according to taxonomic conventions. (+) indicates patients who died within the specified time period (14 days or 30 days), while (−) indicates patients who survived.

**Table 7:** Temporal distribution of *Candida* species according to year of hospitalization.

Candida Species	Year of Hospitalization					*p*-Values	
	2015–2019 (n:151)			2020–2025 (n:240)			
	n	%		n	%		
*C. albicans*	66	43.7%		80	33.3%	**0.039**	χ^2^
*C. parapsilosis*	41	27.2%		51	21.3%	0.180	χ^2^
*Candida* spp.	13	8.6%		44	18.3%	**0.008**	χ^2^
*C. tropicalis*	14	9.3%		20	8.3%	0.749	χ^2^
*C. glabrata*	7	4.6%		25	10.4%	**0.042**	χ^2^
*C. kefyr*	1	0.7%		6	2.5%	0.182	χ^2^
*C. guilliermondii*	1	0.7%		5	2.1%	0.266	χ^2^
*C. ciferrii*	0	0.0%		4	1.7%	0.162	χ^2^
*C. famata*	4	2.6%		0	0.0%	**0.022**	χ^2^
*C. dubliensis*	2	1.3%		0	0.0%	0.149	χ^2^
*C. lusitaniae*	1	0.7%		1	0.4%	1.000	χ^2^
*C. sphaerica*	0	0.0%		2	0.8%	0.525	χ^2^
*C. krusei*	0	0.0%		1	0.4%	1.000	χ^2^
*C. lipolytica*	1	0.7%		0	0.0%	0.386	χ^2^
*C. viswanathii*	0	0.0%		1	0.4%	1.000	χ^2^

Data are presented as numbers (percentages). Comparisons between periods were performed using the chi-square test or Fisher’s exact test, as appropriate. χ^2^ indicates the chi-square test. Bold values indicate statistically significant results (*p* < 0.05).

## Data Availability

The datasets generated and/or analyzed during the current study are not publicly available due to institutional and ethical restrictions related to patient confidentiality but are available from the corresponding author upon reasonable request.

## Abbreviations

The following abbreviations are used in this manuscript:

CVC	Central venous catheter
ICU	Intensive care unit
CRP	C-reactive protein
PCT	Procalcitonin
TPN	Total parenteral nutrition
AST	Aspartate aminotransferase
ALT	Alanine aminotransferase
CLSI	Clinical and Laboratory Standards Institute
ECOFF	Epidemiological cutoff value
OR	Odds ratio
CI	Confidence interval
WBC	White blood cell count
